# Disease-Modifying Anti-rheumatic Drug Prescription Baihu-Guizhi Decoction Attenuates Rheumatoid Arthritis via Suppressing Toll-Like Receptor 4-mediated NLRP3 Inflammasome Activation

**DOI:** 10.3389/fphar.2021.743086

**Published:** 2021-10-05

**Authors:** Weijie Li, Xia Mao, Xiaoyue Wang, Yudong Liu, Kexin Wang, Congchong Li, Taixian Li, Yanqiong Zhang, Na Lin

**Affiliations:** Institute of Chinese Materia Medica, China Academy of Chinese Medical Sciences, Beijing, China

**Keywords:** rheumatoid arthritis, Baihu-Guizhi decoction, balance of the “inflammation-immune” system, pyroptosis, TLR4-NLRP3 inflammasome signaling

## Abstract

As a traditional Chinese medicine-originated disease-modifying anti-rheumatic drug prescription, Baihu-Guizhi decoction (BHGZD) is extensively used for the treatment of rheumatoid arthritis (RA) with a satisfying therapeutic efficacy. Mechanically, our previous data indicated that BHGZD may ameliorate RA partially by restoring the balance of the “inflammation-immune” system through regulating the *TLR4-c-Fos-IL2-TNF-alpha* axis. Toll-like receptor 4 (TLR4) has been revealed to be involved in the activation of the NLR family pyrin domain containing 3 (NLRP3) inflammasome complex. Thus, the aim of the current study was to determine the regulatory effects of BHGZD on the TLR4–mediated inflammasome activation during RA progression based on the modified adjuvant-induced arthritis model (AIA-M) and the lipopolysaccharide/adenosine triphosphate (LPS/ATP)–induced pyroptosis cellular models. As a result, oral administration of BHGZD exhibited prominent improvement in the disease severity of AIA-M rats, such as reducing the redness and swelling of joints, arthritis incidence, arthritic scores, and diameter of the limb and increasing pain thresholds. In line with the *in vivo* findings, BHGZD treatment effectively inhibited the LPS/ATP–induced pyroptosis of both Raw264.7 macrophage and MH7A cells *in vitro* by reducing pyroptotic cell death morphology (swollen cells) and decreasing propidium iodide–positive and terminal deoxynucleotidyl transferase–mediated dUTP-fluorescein nick end labeling (TUNEL)–positive cells. Notably, the increased expression levels of TLR4, NLRP3, interleukin 1β, and interleukin 18 proteins and the elevated activities of caspase-1 and lactic dehydrogenase in *in vivo* and *in vitro* disease models were markedly reversed by the treatment with BHGZD. In conclusion, the above findings proved the immunomodulatory and anti-inflammatory activities of BHGZD, especially in pyroptosis, which may be attributed to the activation of TLR4–mediated NLRP3 inflammasome signaling.

## Introduction

Rheumatoid arthritis (RA), a common systemic autoimmune disease involving multiple organs, is characterized by persistent synovitis, systemic inflammation, and progressive destruction of the cartilage, joint, and bone (Scott, Wolfe, & Huizinga, 2010). RA affects 0.5–1.0% of adults worldwide annually (Gabriel & Michaud, 2009). RA at the active stage (active RA) is characterized by hyperactive immune response and excessive inflammatory cytokines (Mackiewicz, Schooltink, Heinrich, & Rose-John, 1992; ; Wang et al., 2012), including interleukin 1 (IL-1), interleukin 6 (IL-6), and interleukin 17 (IL-17) exerting their influences on both osteoclast differentiation and osteoblasts and active joint inflammation (Kawanaka et al., 2002; Dharmapatni et al., 2009; Karmakar et al., 2010). The key therapeutic agents, including disease-modifying antirheumatic drugs (DMARDs), nonsteroidal anti-inflammatory drugs (NSAIDs), glucocorticoids, and biological response modifiers, reduce synovitis and systemic inflammation and improve their function (Kirwan, 1995). Among them, methotrexate (MTX), one of the DMARDs, is indicated for severe active RA. MTX has been used in the treatment of RA since the 1980s and is commonly used as the first-line medication for RA treatment (Li W. et al., 2020). However, poor patient’s response, infection, and high costs often restrict the prescription of this drug (Urushibara et al., 2004; Andersson et al., 2008; Aletaha et al., 2017). Therefore, it is an urgent necessity to explore and identify alternative therapeutic strategies for RA treatment.

Traditional Chinese medicine (TCM) has its unique advantages in treating complex and chronic diseases, especially in the clinical treatment of active RA (Zhang et al., 2010). Baihu-Guizhi decoction (BHGZD), a TCM–originated DMARD prescription, is recorded by a Chinese medical sage Zhang Zhongjing in Jin Kui Yao Lue and consists of Gypsum (sulfates; Gypsum Fibrosum), *Anemarrhena asphodeloides* Bge. (Liliaceae; Anemarrhenae Rhizoma), *Cinnamomum cassia* Presl (Lauraceae; Cinnamomi Ramulus), *Oryza sativa* L. (Gramineae; Oryza Semen), and *Glycyrrhiza uralensis* Fisch (Leguminosae; Glycyrrhizae Radix et Rhizoma). Among them, *Oryza sativa* L. and *Glycyrrhiza* uralensis Fisch are renowned for their homology of medicine and food superiority in traditional and alternative medicine, having a high research value.

Accumulated clinical trials have revealed that BHGZD achieves satisfactory therapeutic response in the treatment of active RA, alleviating symptoms and signs of the disease such as pain, morning stiffness, joint tenderness, swelling, and deformity, as well as levels of rheumatoid factor (RF), C-reactive protein (CRP), and erythrocyte sedimentation rate (ESR) especially in RA patients with wind–damp–heat stimulation with a clinical efficiency as high as 90% (Yuan et al., 2019; Wu, 2021). In addition, the mean DAS28 score of RA patients decreases after BHGZD treatment (DAS28 score: 5.49 to 2.93) (Yuan, et al., 2019). BHGZD significantly ameliorates infectious mononucleosis *via* decreasing the levels of CD3^+^, CD8^+^, aspartate aminotransferase (AST), lactic dehydrogenase (LDH), creatine (9CK), and isoenzyme (CK-MB) and increasing the levels of CD4^+^, CD4+/CD8+, and IgA and IgG (Zhang C. et al., 2016). Previously, our group, combining the chemical and transcriptomic profiling, target prediction, network calculation, and experimental validations, identified the chemical constituents contained in BHGZD and revealed that BHGZD may ameliorate RA partially by restoring the balance of the “inflammation-immune” system and subsequently reversing the pathological events during RA progression through regulating the *TLR4-c-Fos-IL2-TNF-alpha* axis (Li W. et al., 2020), which was in line with the findings of other research groups (Li et al., 2017; Fang, 2018).

Pyroptosis is a highly regulated process of cell death, which is essential for physiological processes such as organ development, cell renewal, and differentiation (Shi et al., 2017). The nucleotide-binding oligomerization domain-like receptor protein 3 (NLRP3) inflammasome composed of NLRP3 proteins, apoptosis-associated speck-like proteins containing a caspase recruitment domain (ASC), and caspase-1 have been extensively studied (Martinon et al., 2002; Ting et al., 2008; Zeng et al., 2019). Increasing evidence showed that NLRP3 inflammasome may be involved in the pathogenesis of RA (Guo et al., 2018; Kolly et al., 2010). NLRP3 may be activated by TLR4 signaling (Chi et al., 2015; Martin-Rodriguez et al., 2015), resulting in the formation of intracellular inflammasome complexes with the adaptor protein ASC and caspase-1–dependent cleavage and secretion of interleukin 1 beta (IL-1β) (Netea et al., 2008; Kate and jurg, 2010; Strowig et al., 2012; Zhang H. et al., 2016), which is a well-characterized outcome of TLR and inflammasome cooperation (Chi et al., 2015). In the current study, we investigated the regulatory effects of BHGZD on TLR4–mediated NLRP3 inflammasome activation during RA progression based on the modified adjuvant-induced arthritis model (AIA-M) as well as Raw264.7 macrophage and MH7A cells.

## Materials and Methods

### Ethics Statement

The study was approved by the Research Ethics Committee of the Institute of Basic Theory of Chinese Medicine, China Academy of Chinese Medical Sciences, Beijing, China (SYXK 2016-0021, Beijing, China). All animal studies were treated in accordance with the guidelines and regulations for the use and care of animals of the Center for Laboratory Animal Care, China Academy of Chinese Medical Sciences.

### Preparation of Baihu-Guizhi Decoction

Crude drugs of gypsum (60 g), *Anemarrhena asphodeloides* Bge. (15 g), *Cinnamomum cassia* Presl (10 g), *Oryza sativa* L. (30 g), and *Glycyrrhiza uralensis* Fisch (5 g) were purchased from Beijing Tongrentang Co., Ltd. (Beijing, China). BHGZD was prepared according to the original composition of the formula recorded in the Chinese Pharmacopoeia 2020 edition and obtained based on our previous study (Supplementary Material Section 1). The filtrate was combined and concentrated under reduced pressure and dried out in an oven at 70°C overnight to obtain the BHGZD powder.

### Animals

Male Lewis rats (*n* = 51, 6∼8-week-old, 200 ± 20 g in weight) were purchased from Beijing Vital River Laboratory Animal Technology Co., Ltd. (production license no. SCXK 2016-0006, Beijing, China). All animals were maintained under specific pathogen-free conditions in a temperature-controlled room at a constant temperature of 24 ± 1°C with a 12°h light/dark cycle and had free access to the standard rodent diet and water *ad libitum*.

### Grouping and Treatment

A total of 51 rats were randomly divided into four groups: 1) normal control group (*n* = 15); 2) AIA-M (*n* = 15); 3) AIA-M-BHGZD treatment (*n* = 15); and 4) AIA-M-MTX treatment (positive control group, *n* = 6).

Induction of the AIA-M was performed as previously reported (Li W. et al., 2020; Mao et al., 2020), and AIA-M rats were established to simulate the pathological changes and characteristics of active RA after AIA-M induction. In brief, male Lewis rats were injected intradermally through the base of the tail with 10 mg/mL M *tuberculosis* H37 Ra (Difco, BD company, New Jersey, United States) suspended in liquid paraffin (Freund’s complete adjuvant (CFA)). From the day of primary immunization, the male Lewis rats were kept in an artificial climate box (production license no. RXZ-380A-LED) for 2 h daily with certain wind velocity (6 m/s), temperature (37°C), and humidity (90%) for a period of 15 days.

In the AIA-M-BHGZD treatment group, the dosage selection for BHGZD was 21.4 g/kg, nearly equivalent to two times of the daily dosage of RA patients in clinics, which has been proved to exert the most prominent therapeutic efficacy as in our previous study (Li W. et al., 2020). The dosage for MTX was 0.2 mg/kg. All treatments were performed for 30 days *via* oral administration from the day of primary immunization.

### Assessment of Arthritis Severity

The severity of arthritis was evaluated by arthritis accidence, arthritis score, diameter of the limb, and body weight as per our previous studies (Wang et al., 2015; Zhang Y. et al., 2016; Guo et al., 2016; Li W. et al., 2020; ). Detailed information has been given in Supplementary Material Section 2. The temperature of the articular surface was detected as described in Supplementary Material Section 3.

### Measurement of Mechanically, Acetone-, and Thermally Induced Hyperalgesia

The pain threshold was evaluated by mechanically, acetone-, and thermally induced hyperalgesia as described previously (Wang et al., 2015; Guo et al., 2016; Li W. et al., 2020; Zhang Y. et al., 2016). Detailed information on the protocol is provided in Supplementary Material Sections 4–6.

### Thymus and Spleen Indexes

The weight ratios of the thymus, spleen, liver, and kidney relative to the total brain weight were calculated, as described in Supplementary Material Section 7.

### Cell Culture

Mouse macrophage cell lines (Raw264.7) and immortalized cell lines of synovial fibroblasts from the articular cavity in RA patients (MH7A cells) were used for experiment validation *in vitro*. Raw264.7 macrophage cells were maintained in DMEM (Hyclone, Logan, UT, United States), supplemented with 10% fetal bovine serum (FBS, Gibco, Carlsbad, CA, United States), 100 U/mL penicillin, 100 μg/ml streptomycin, and 2 mML glutamine (TBD, Tianjin, China) in a humidified 5% CO_2_ incubator at the temperature of 37°C. The Raw264.7 macrophage cells of low passage number were used in the current experiment validations.

MH7A cells were cultured in a sterile synoviocyte growth medium (Cell Applications, Inc., San Diego, CA) added with 10% synoviocyte growth supplement (Cell Applications, Inc., San Diego, CA), 100 U/mL penicillin, 100 μg/ml streptomycin, and 2 mML glutamine (TBD, Tianjin, China) in a humidified 5% CO_2_ incubator at the temperature of 37°C. MH7A cells of passage numbers four to eight were used in the current experiment validations.

### NLRP3 Inflammasome Activation

To induce a conventional NLRP3 inflammasome activation, Raw264.7 macrophage or MH7A cells were induced with 0.2 μg/ml or 1 μg/ml lipopolysaccharide [LPS, *Escherichia coli* (O111:B4), Sigma-Aldrich, St Louis, MO, United States] for 4 or 6 h, respectively, followed by adding a 3 mM ATP (adenosine triphosphate) (ATP disodium salt hydrate, Sigma-Aldrich, St Louis, MO, United States) for 1 h and incubation at 37°C/5% CO_2_ (Li et al., 2018; Zhao L. R. et al., 2018), in the presence or absence of the NLRP3 inflammasome inhibitor.

A selective NLRP3 inhibitor MCC950 was purchased from Selleck Chemicals (CP-456773 Sodium, Selleck, Houston, TX, United States). 100nM MCC950 sodium was added for 30 min before NLRP3 stimulation (Huang et al., 2017).

### Enzyme-Linked Immunosorbent Assay

On the 31st day, all rats were anesthetized by intraperitoneal injection of pentobarbital sodium (50 mg/kg). The rats were secured in the supine position, and the blood was taken from the abdominal aorta using one-time anticoagulant negative pressure blood collection tubes. The blood was placed at room temperature for 20 min and centrifuged at 12,000 rpm at low temperature (4°C) for 15 min. The supernatant was collected, vortexed, and centrifuged again at high speed for 15 min. The cells were centrifuged, and supernatants were collected.

The expression levels of TLR4, IL-1β, and IL-18 proteins and activity of caspase-1 in the sera of AIA-M rats, as well as IL-1β and IL-18 in culture supernatants, were estimated using the ELISA kit (ML Bio, Shanghai, China) according to the manufacturer’s instructions. The absorbance was measured using a Multiskan™ GO microplate spectrophotometer (Thermo Fisher Scientific, Waltham, MA, United States). Detailed information on the ELISA kit used is listed in Supplementary Material Table S1.

### Lactic Dehydrogenase Assay

Pyroptosis was assessed by LDH release. LDH release in the sera and culture supernatant were assayed using the LDH ELISA kit (ML Bio, Shanghai, China), according to manufacturer’s instructions. The absorbance was measured using a Multiskan™ GO microplate spectrophotometer (Thermo Fisher Scientific, Waltham, MA, United States). Detailed information on the ELISA kit used is listed in Supplementary Material Table S1.

### Western Blotting

To evaluate the regulatory activities of BHGZD on the candidate therapeutic targets in the arthritic tissue sample and Raw264.7 macrophage and MH7A cells treated with drugs, western blotting analysis was performed following the protocol of our previous studies (Li W. et al., 2020). The following antibodies were used: TLR4 (rabbit anti-TLR4 antibody, dilution 1:1,000, Bioss Antibodies, Beijing, China), NLRP3 (NLRP3 rabbit antibody, dilution 1:1,000, ABclonal Technology, Wuhan, China), ASC (ASC antibody, dilution 1:1,000, Santa Cruz Biotechnology, Santa Cruz, CA, United States), caspase-1 (caspase-1 (D7F10) rabbit antibody, dilution 1:1,000, Cell Signaling Technology, Danvers, Massachusetts, United States), GSDMD (recombinant anti-DFNA5/GSDME antibody-N-terminal, dilution 1:1,000, Abcam, Cambridge, United Kingdom), and IL-1β (IL-1beta rabbit antibody, dilution 1:1,000, ABclonal Technology, Wuhan, China). The mean normalized protein expression±S.D. was calculated from independent experiments. GAPDH (GAPDH polyclonal antibody, dilution 1:10,000, Proteintech, Chicago, United States) and β-actin (anti-beta-actin/β-actin antibody, dilution 1:2000, Boster Biological Technology, California, United States) were used as loading controls of arthritic tissue samples and cultured cells, respectively.

### Immunofluorescence

For NLRP3/ASC double immunofluorescence, rabbit anti-NLRP3 (dilution 1:100, ABclonal Technology, Wuhan, China) and mouse anti-ASC (dilution 1:50, Santa Cruz Biotechnology, Santa Cruz, CA, United States) primary antibodies were used. Sections were then labeled with FITC– and Cy3–conjugated secondary antibodies (dilution 1:200, Servicebio, Wuhan, China) for 2 h at room temperature, avoiding light, followed by counterstaining with 4′,6-diamidino-2-phenylindole (DAPI) staining solution (Servicebio, Wuhan, China). Subsequently, the anti-fluorescence quencher was used to seal the section (Servicebio, Wuhan, China). Fluorescence images were photographed with a Zeiss LSM 880 confocal microscope (Carl Zeiss, Jena, Germany).

### Caspase-1 Activity

Active caspase-1 was visualized with a FAM-FLICA caspase-1 assay kit using the FAM-YVAD-FMK inhibitor probe (ImmunoChemistry Technologies, Bloomington, MN, United States), according to manufacturer’s guidelines. Fluorescence images were photographed with a Zeiss LSM 880 confocal microscope (Carl Zeiss, Jena, Germany).

### Terminal Deoxynucleotidyl Transferase–Mediated dUTP-Nick End Labeling Assay

Cell apoptosis was determined using a TUNEL Andy Fluor™ 594 Apoptosis Detection kit (ABP Bioscience, Wuhan, China), in accordance with the manufacturer’s protocol. Fluorescence images were photographed with an inverted fluorescence microscope (MSHOT, Guangzhou, China).

### Flow Cytometry

After treatment, Raw264.7 macrophage cells were double-stained with Annexin V-fluorescein isothiocyanate and propidium iodide (PI) (FITC Apoptosis Detection Kit I; BD Biosciences, San Jose, CA, United States) according to the instructions. Quantification was then performed using a flow cytometer (NovoCyte 2040R; ACEA Bioscience, San Diego, CA, United States) and analyzed using NovoExpress 1.4.1 Software (ACEA Bioscience, San Diego, CA, United States).

### Statistical Analyses

Statistical analyses were performed using GraphPad Prism 8.0 Software (San Diego, CA, United States). Data are expressed as the mean ± S.D. and were analyzed by one-way ANOVA with Bonferroni’s or Dunnett’s post hoc test for comparison of multiple columns. Differences were considered statistically significant when the *p* value was less than 0.05.

## Results

### Baihu-Guizhi Decoction Treatment Alleviates Disease Severity of Arthritis in Adjuvant-Induced Arthritis Model Rats

**FIGURE 1:**
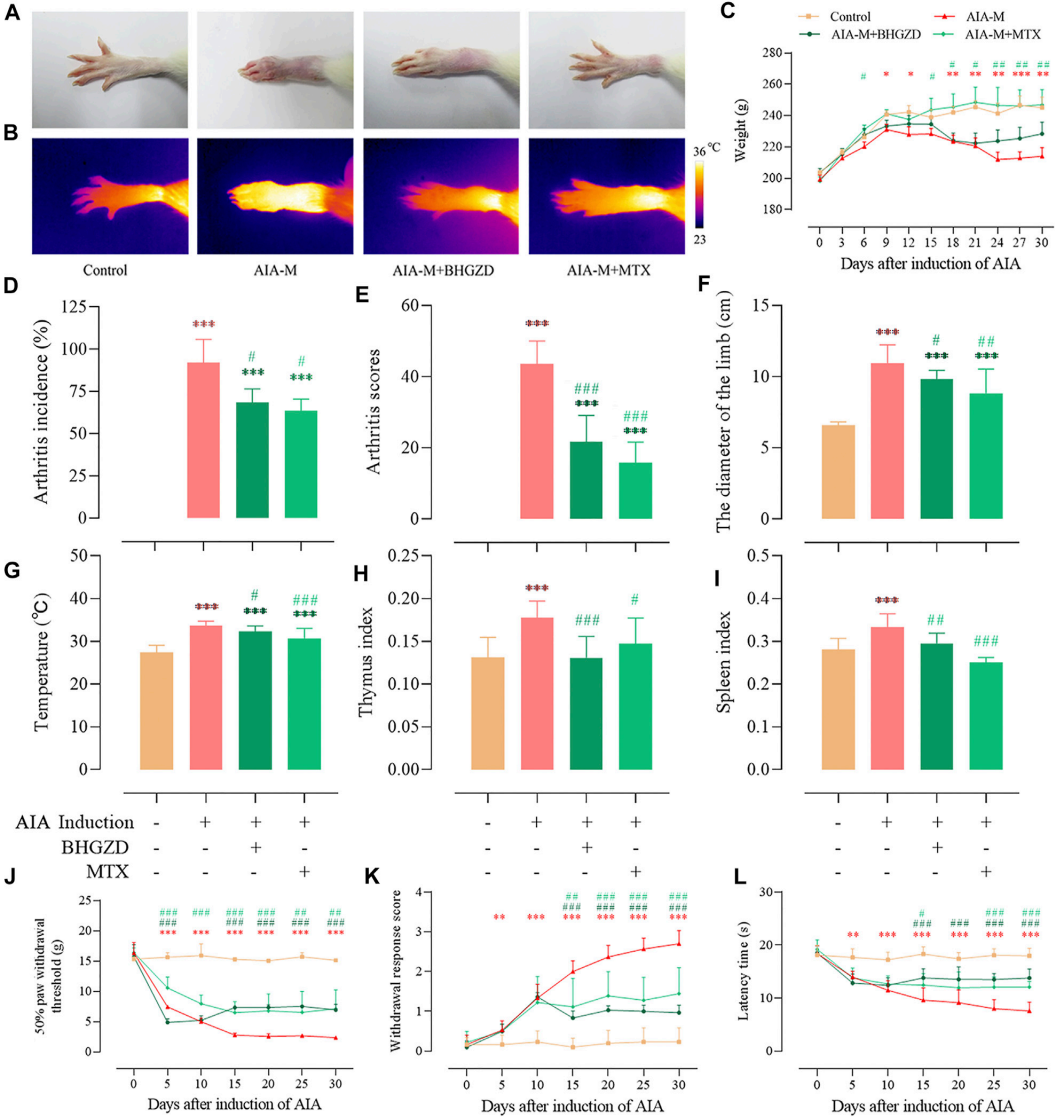
Effects of BHGZD on the severity of arthritis in AIA-M rats of different groups. **(A)** Macroscopic performances of arthritis; **(B)** infrared thermography; **(C)** weight; **(D)** arthritis incidence; **(E)** arthritis scores; **(F)** the diameter of the limb; **(G)** articular temperature; **(H)** the thymus index; **(I)** the spleen index; **(J)** mechanically induced hyperalgesia; **(K)** acetone-induced hyperalgesia; **(L)** thermally induced hyperalgesia. Data are expressed as the mean ± S.D. *, **, and ***, *p* < 0.05, *p* < 0.01, and *p* < 0.001, respectively, in comparison with the normal control group; #, ##, and ###, *p* < 0.05, *p* < 0.01, and *p* < 0.001, respectively, in comparison with the AIA-M group.

To determine the pharmacological effects of BHGZD against AIA-M, we successfully established AIA-M rats simulating pathological changes and characteristics of RA with severe redness and swelling (Figure 1A). The final incidence (incidence on the 25th day after the first immunization), mean arthritis score, diameter of the limb, and articular temperature were approximately 100%, 43.56, 10.95 cm, and 33.65°C, respectively (Figure 1D∼F). BHGZD treatment strikingly improved the severity of arthritis, including the reduced arthritis incidence (*p* < 0.05, Figure 1D), arthritis scores (*p* < 0.001, Figure 1E), diameter of the limb (*p* < 0.05, Figure 1F), and articular temperature (*p* < 0.05, Figure 1G), which were consistent with the macroscopic evidence of arthritis (Figure 1A).

In terms of the response to inflammation, the thymus and spleen indexes in different groups were determined (Figure 1H∼I). BHGZD treatment decreased the thymus and spleen indexes of AIA-M rats significantly.

Simultaneously, the body weight (Figure 1C) and pain thresholds (mechanically, acetone-, and thermally induced hyperalgesia) in AIA-M rats were significantly elevated when treated with BHGZD (all *p* < 0.05, Figure 1J∼K). The pharmacological effects of BHGZD were similar to that of the positive drug MTX. No hepatic or renal toxicities were observed after BHGZD treatment (Supplementary Material Figure S1).

### Baihu-Guizhi Decoction Suppresses TLR4–Mediated NLRP3 Inflammasome Activation in Adjuvant-Induced Arthritis Model Rats

**FIGURE 2:**
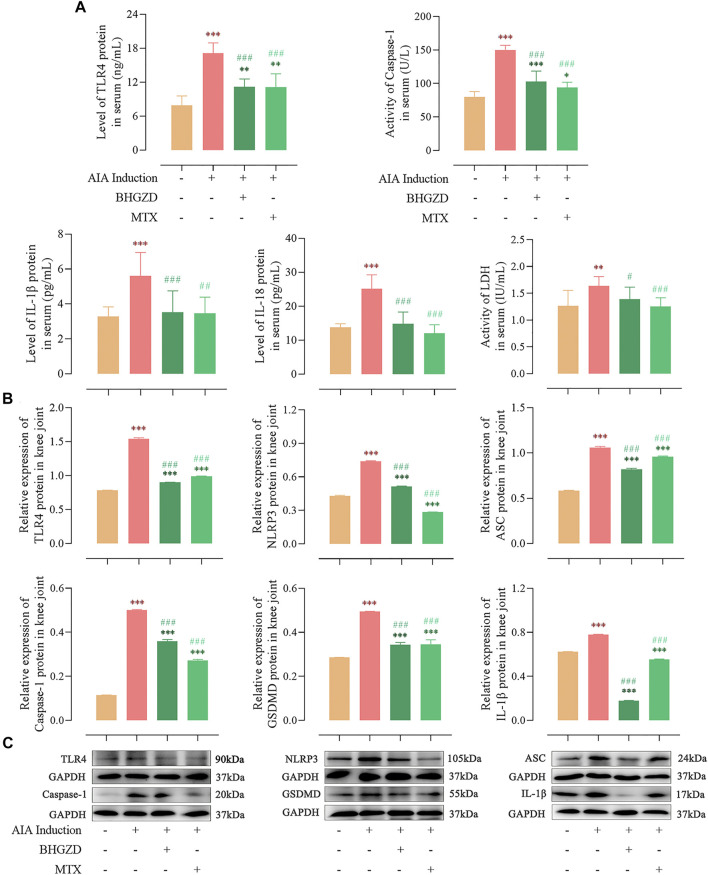
Effects of BHGZD treatment on the expression of proteins of TLR4–mediated NLRP3 inflammasome in AIA-M rats of different groups detected by ELISA and Western blotting. **(A)** Expression levels of TLR4, IL-1β, and IL-18 proteins and activities of caspase-1 and LDH in the sera of AIA-M rats of different groups. **(B ∼ C)** Protein expression levels of TLR4, NLRP3, ASC, caspase-1, GSDMD, and IL-1β in the joints of AIA-M rats of different groups. *, **, and ***, *p* < 0.05, *p* < 0.01, and *p* < 0.001, respectively, in comparison with the normal control group; #, ##, and ###, *p* < 0.05, *p* < 0.01, and *p* < 0.001, respectively, in comparison with the AIA-M group.

Consistent with our previous study (Li W. et al., 2020), the expression of TLR4 proteins was significantly higher in both sera and the knee joint of AIA-M rats, which was reduced by the treatment with BHGZD (all *p* < 0.001, Figure 2).

To address the regulation of BHGZD on NLRP3 inflammasome activation, the expression levels of the NLRP3 inflammasome pathway were detected by ELISA and Western blotting, respectively. The protein expression levels of NLRP3, ASC, and caspase-1 p20 (the active form of caspase-1) were markedly increased, which were decreased by the treatment with BHGZD (all *p* < 0.001, Figure 2). In addition, the activity of caspase-1 in sera was detected by ELISA, which demonstrated the reduced effect of BHGZD on the activity of caspase-1. Notably, we found that the protein expression levels of GSDMD and inflammatory cytokines (IL-1β and IL-18) were markedly increased, which were reduced by the treatment with BHGZD (*p* < 0.001, Figure 2). Moreover, the LDH activity was used to assess pore formation and release of intracellular soluble components. The enhancing activity of LDH in the sera of AIA-M rats was observed, which was decreased by BHGZD treatment (*p* < 0.05, Figure 2A).

### Baihu-Guizhi Decoction Suppresses Lipopolysaccharide/Adenosine Triphosphate–Induced Pyroptosis in Raw264.7 Macrophage and MH7A Cells

To verify our *in vivo* findings based on AIA-M rats, an established method (LPS plus ATP) was applied to induce NLRP3 inflammasome activation in both Raw264.7 macrophage and MH7A cells. We initially evaluated the cytotoxicity of BHGZD on the growth of Raw264.7 macrophage cells using flow cytometry analysis, exhibiting no cell toxicity under BHGZD treatments of 7.14, 14.27, and 28.54 μg/ml, as shown in Supplementary Material Figure S2. Thus, the low, middle, and high doses of BHGZD treatment were chosen in the following assays.

**FIGURE 3:**
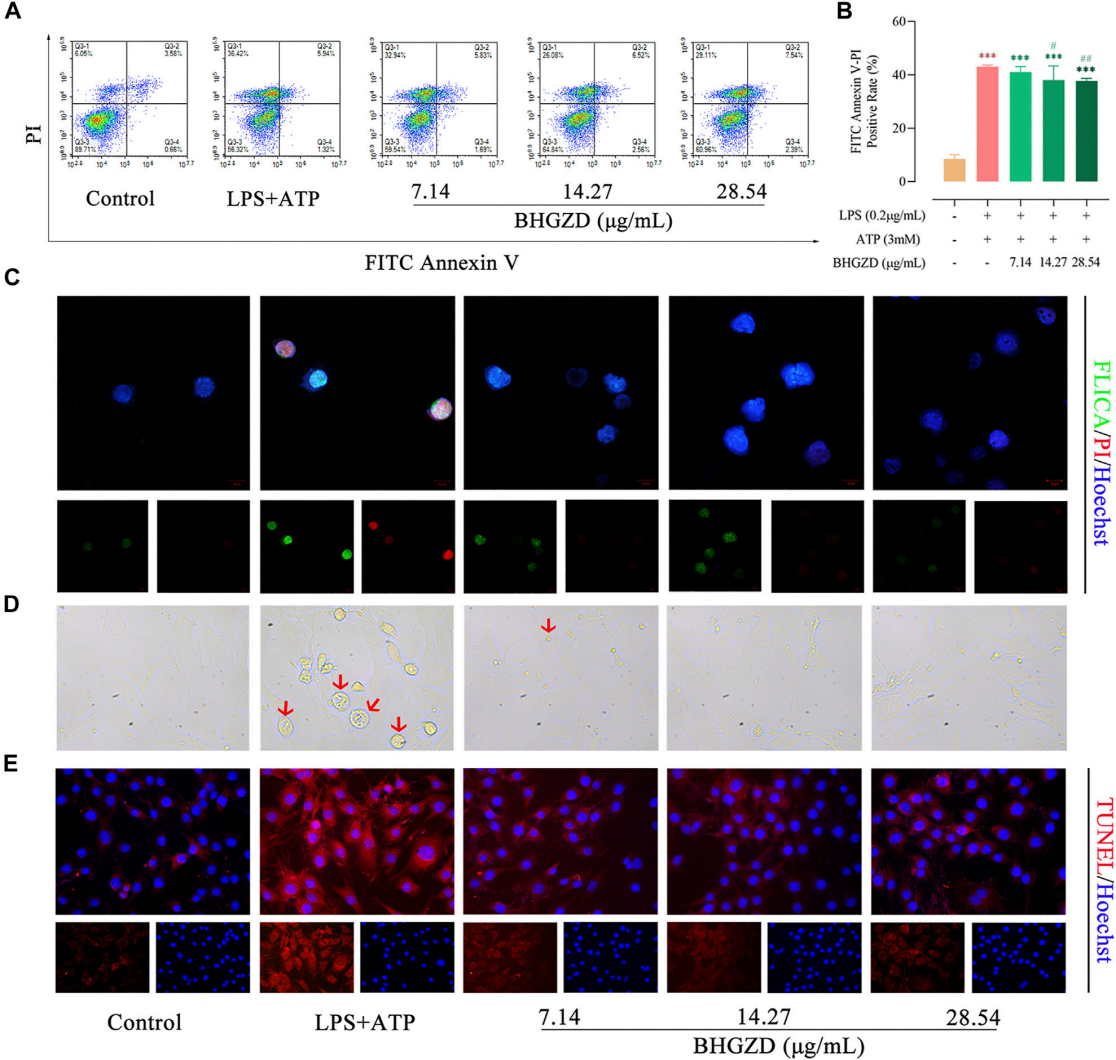
BHGZD inhibits LPS/ATP–induced pyroptosis in both Raw264.7 macrophage and MH7A cells. **(A ∼ B)** Flow cytometry analysis for Annexin V/PI staining in Raw264.7 macrophage cells. **(C)** Representative images of FLICA caspase-1 that binds only to activated caspase-1 (scale bar represents 10 μm; FAM-FLICA green, PI red, Hoechst blue). **(D)** Representative phase-contrast images of MH7A cells (scale bar represents 50 μm). Arrows indicate cells undergoing pyroptosis (swollen cells). **(E)** TUNEL staining of MH7A cells (scale bar represents 50 μm; TUNEL red, Hoechst blue). ***, *p* < 0.001, in comparison with the normal control group; # and ##, *p* < 0.05 and *p* < 0.01, respectively, in comparison with the LPS/ATP–induced model.

The aggravated pyroptotic cell death morphology in cultured Raw264.7 macrophage and MH7A cells (membrane swell, Figure 3D) was observed, which was reversed by the treatment with BHGZD in different doses (7.14, 14.27, and 28.54 μg/ml). Then, flow cytometry analysis revealed that BHGZD treatment prominently reduced the PI–positive cell rate [a marker of cells that stains necrotic, dead, and membrane-compromised cells (Yang J. et al., 2016)], indicating the amelioration of cell membrane pore formation induced by LPS/ATP (Figure 3A∼B). In addition, the co-staining with FAM-FLICA caspase-1 and PI was performed to visualize pyroptosis. Importantly, activation of caspase-1 was observed in LPS/ATP–induced Raw264.7 macrophage cells exposed to radiation by FAM-FLICA caspase-1 staining, which was suppressed by the treatment with BHGZD (Figure 3C). Moreover, BHGZD apparently decreased TUNEL–positive cells in LPS/ATP–induced MH7A cells (Figure 3E).

### Baihu-Guizhi Decoction Suppresses the Activation of the NLRP3 Inflammasome in Raw264.7 Macrophage and MH7A Cells

**FIGURE 4:**
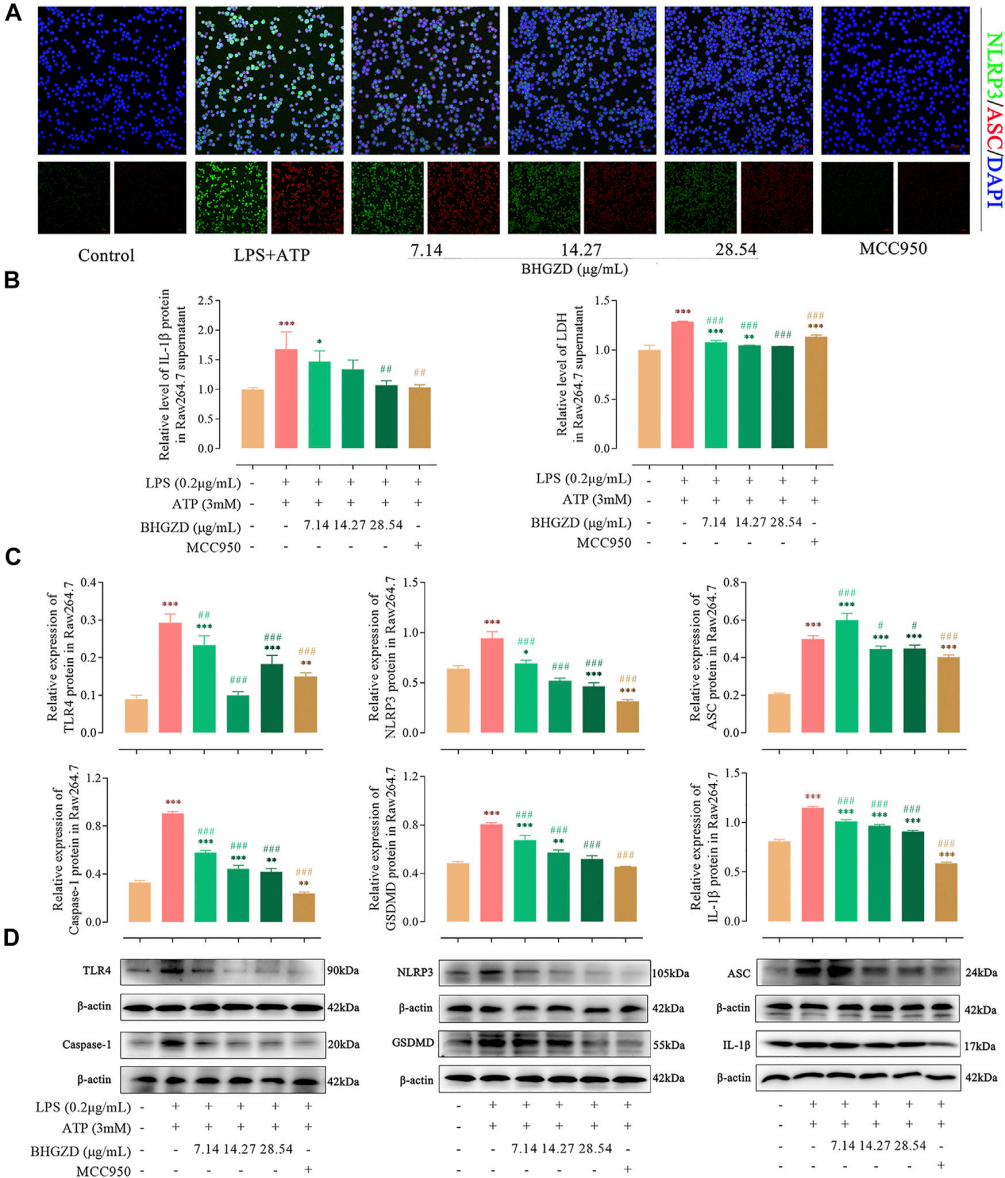
Inhibitory effects of BHGZD on the TLR4–mediated NLRP3 inflammasome activation in LPS/ATP–induced Raw264.7 macrophage cells. **(A)** The expression of NLRP3 and ASC proteins was measured by immunofluorescence staining and confocal microscopy in Raw264.7 macrophage cells (NLRP3 FITC green, ASC CY3 red, DAPI blue; scale bar represents 50 μm). **(B)** The secretion of IL-1β and LDH release in Raw264.7 macrophage cells were evaluated by ELISA. **(C ∼ D)** The expression levels of TLR4, NLRP3, ASC, caspase-1, GSDMD, and IL-1β in Raw264.7 macrophage cells were measured by Western blotting. *, **, and ***, *p* < 0.05, *p* < 0.01, and *p* < 0.001, respectively, in comparison with the normal control group; #, ##, and ###, *p* < 0.05, *p* < 0.01, and *p* < 0.001, respectively, in comparison with LPS/ATP–induced model.

**FIGURE 5:**
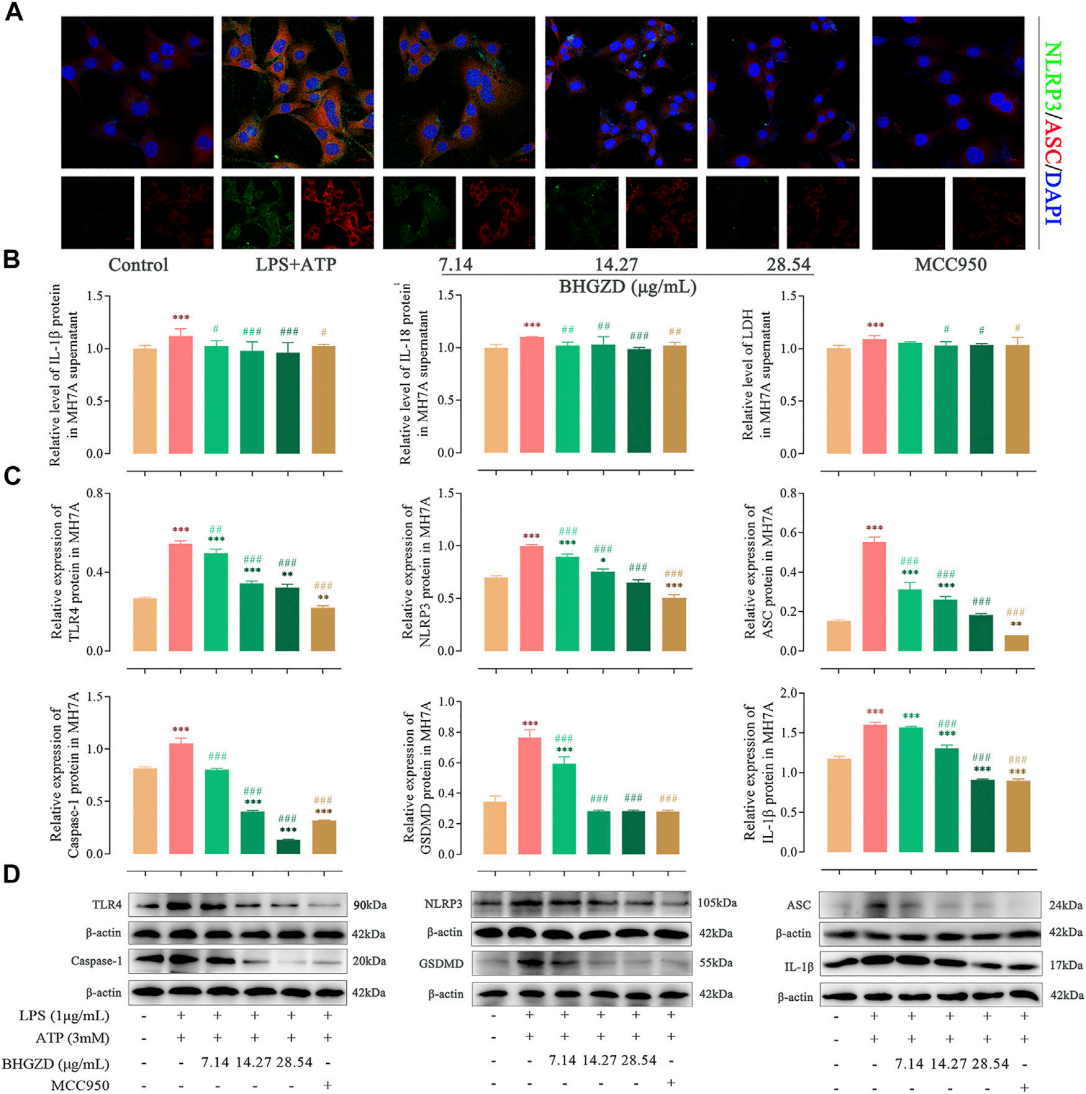
Inhibitory effects of BHGZD on the TLR4–mediated NLRP3 inflammasome activation in LPS/ATP–induced MH7A cells. **(A)** The expression of NLRP3 and ASC proteins was measured by immunofluorescence staining and confocal microscopy in MH7A cells (NLRP3 FITC green, ASC CY3 red, DAPI blue; scale bar represents 20 μm). **(B)** The secretions of IL-1β, IL-18, and LDH release in MH7A cells were evaluated by ELISA. **(C ∼ D)** The expression levels of TLR4, NLRP3, ASC, caspase-1, GSDMD, and IL-1β in MH7A cells were measured by Western blotting. *, **, and ***, *p* < 0.05, *p* < 0.01, and *p* < 0.001, respectively, in comparison with the normal control group; #, ##, and ###, *p* < 0.05, *p* < 0.01, and *p* < 0.001, respectively, in comparison with the LPS/ATP–induced model group.

To further explore the mechanism of BHGZD against pyroptosis, the expression levels of the proteins related to the NLRP3 inflammasome pathway were detected by immunofluorescence staining, ELISA, and Western blotting. The immunofluorescence staining result showed that the co-expression level of NLRP3 and ASC was increased, which was significantly reduced by the treatment with BHGZD in both Raw264.7 macrophage and MH7A cells, similar to the effects of MCC950 (Figure 4A and Figure 5A). As shown in Figure 4B∼D, BHGZD treatment decreased the expression levels of IL-1β proteins in both cultured cells and supernatants and LDH release in LPS/ATP–induced Raw264.7 macrophage cells (all *p* < 0.05 in 28.54 μg/ml BHGZD). Consistent with the data, BHGZD treatment decreased the expression levels of IL-1β proteins in both cultured cells and supernatants, as well as the level of IL-18 and LDH release in MH7A cell supernatants (all *p* < 0.05 in 14.27 and 28.54 μg/ml BHGZD treatment, Figure 5B∼D). As the maturation of IL-1β results from NLRP3 inflammasome activation, which was characterized by decreased protein expression levels of NLRP3, ASC, and caspase-1 when treated with BHGZD in LPS/ATP–induced Raw264.7 macrophage and MH7A cells (Figure 4C∼D and Figure 5C∼D).

In addition, LPS/ATP induction profoundly increased the expression of TLR4 proteins, while BHGZD treatment significantly reduced the TLR4 protein expression (all *p* < 0.05, Figure 4C∼D and Figure 5C∼D), revealing that BHGZD suppressed toll-like signaling activation. Moreover, Western blotting results showed that GSDMD occurred in the LPS/ATP–induced group and was decreased by the treatment with BHGZD (all *p* < 0.001, Figure 4C∼D and Figure 5C∼D).

## Discussion

Increasing clinical evidence shows that BHGZD may be highly applied to most patients with RA during the active phase representing the damp–heat impeding syndrome (Wang et al., 2012), featured by severe immune response and inflammatory reaction (Latz et al., 2013;; Wang et al., 2012). Previously, we indicated that BHGZD reversed the imbalance of the “inflammation-immune” system by suppressing TLR4 activation (Li W. et al., 2020). Accumulated studies have demonstrated that TLR4 initiates NLRP3 inflammasome–mediated pyroptosis (Chi et al., 2015; Martin-Rodriguez et al., 2015; Yin et al., 2020; X.; Zhang et al., 2020), which is a type of programmed cell death (Hersoug et al., 2018), largely involved in the pathogenesis of cell death, liver fibrosis (Wree et al., 2014), and chronic and acute inflammation (So et al., 2013; Zamyatina & Heine, 2020), such as RA (Guo et al., 2018; Li Z. et al., 2020; Pope & Tschopp, 2007; Shen et al., 2018; Wu et al., 2020). Especially, high expression levels of NLRP3 mRNA and NLRP3 inflammasome protein were observed in monocytes/macrophages, fibroblast-like synoviocytes (FLS), dendritic cells, and neutrophils from RA patients (Choulaki et al., 2015; Kim et al., 2017; Ruscitti et al., 2015;; Yang Z. et al., 2016). In the current study, our data based on the *in vivo* and *in vitro* experiments demonstrated for the first time the regulatory effects of BHGZD on the TLR4–mediated pyroptosis, which may underlie the main pathological changes during active RA (Wu et al., 2020).

In the current study, *in vivo* experimental data showed that BHGZD treatment significantly ameliorated the disease severity of arthritis in AIA-M rats, including the prominent improvement of redness and swelling of joints, arthritis scores, diameter of the limb, articular temperature, body weight, and pain thresholds. In addition, the thymus and spleen indexes of AIA-M rats were increased by the treatment with BHGZD. The spleen and thymus, central immune organs, are the main places for immune cell development, maturation, differentiation, and recruitment (Res & Spits, 1999). The values of spleen and thymus indexes reflect and affect the immune function in the body. The above data suggest that BHGZD may regulate the immune reaction in the progression of active RA, in line with our previous findings that BHGZD treatment typically preserved the articular cartilage matrix integrity in markedly inflamed joints (Li W. et al., 2020). In addition, our previous study verified that the anti-inflammatory and immunomodulatory effects of BHGZD were achieved through suppressing TLR4 activation (Li W. et al., 2020). Accordingly, we focused on determining the regulatory effects of BHGZD on the TLR4–mediated pyroptosis using the LPS/ATP–induced pyroptosis cellular models. As a result, BHGZD treatment significantly improved pyroptotic cell death morphology (swollen cells), decreased PI– and TUNEL–positive cells, and suppressed caspase-1 activation. RA involves erosions in the marginal bone along with the articular cartilage, leading to joint destruction. The process of pyroptosis is linked with the destruction of the plasma membrane, which releases cytokines, and excessive release of other mediators (Chadha et al., 2020; Wu et al., 2020). Active caspase-1 formation is associated with pyroptosis and inflammasome activity, and active caspase-1 takes on the role of a key housekeeping enzyme in its conversion of pro-IL-1β proteins into the active IL-1β cytokine and is measured *via* irreversible binding of a probe composed of an inhibitor–peptide sequence containing a fluorescent tag, referred to as FAM-FLICA (Fernandez et al., 2016). Pyroptosis is a type of programmed cell death with several features including DNA damage detected by the TUNEL assay (Miao et al., 2011). Similar to apoptotic cells, the pyroptotic cells were PI– or TUNEL–positive (Labbe & Saleh, 2008). These results clearly indicated that BHGZD significantly inhibited pyroptotic death through suppressing TLR4–mediated NLRP3 inflammasome activation.

**FIGURE 6:**
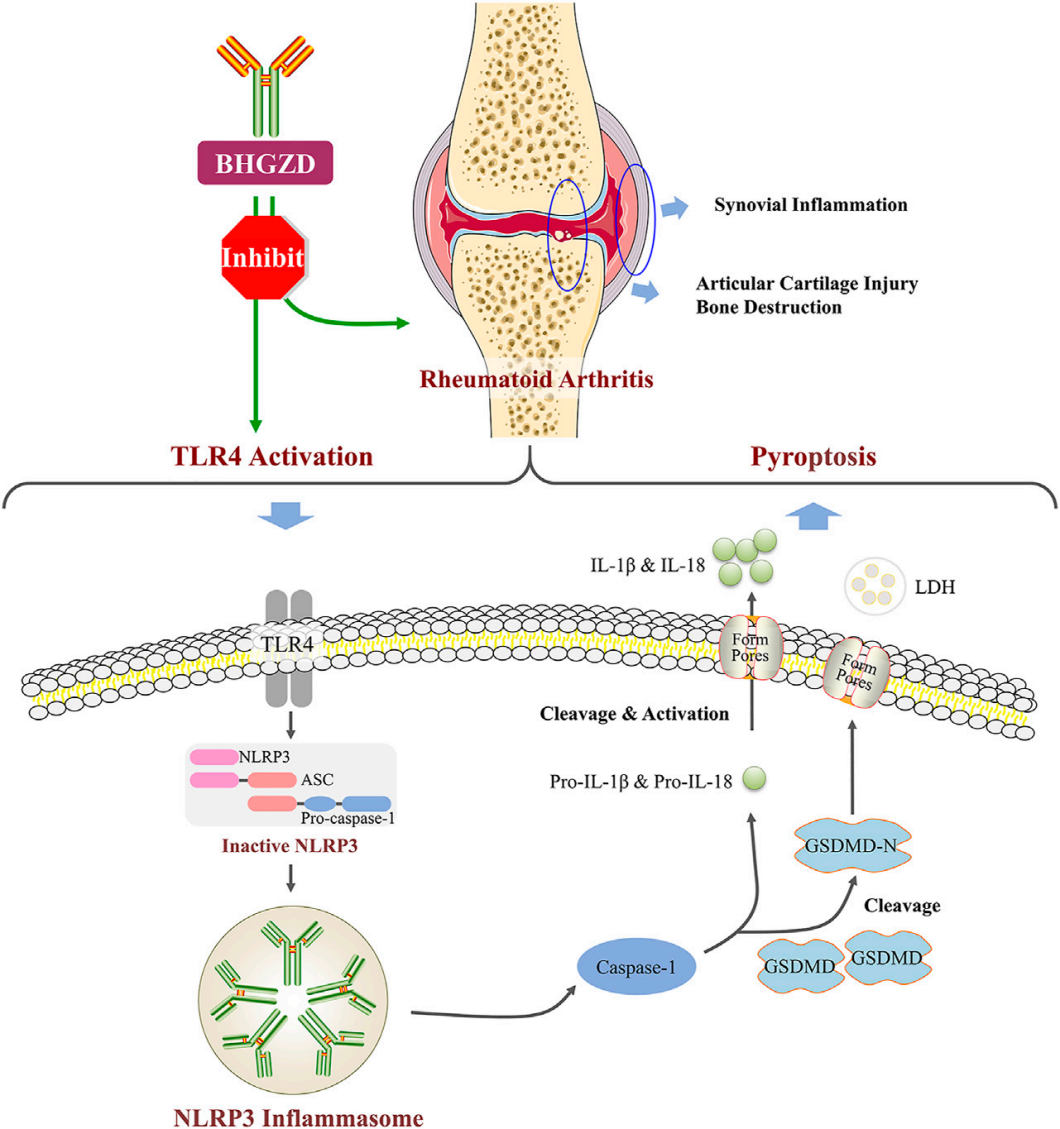
BHGZD attenuates RA *via* suppressing TLR4–mediated NLRP3 inflammasome activation.

It is well known that chronic inflammatory response may be the mainly protracted course of RA disease. We further confirmed that BHGZD inhibited the TLR4–mediated inflammasome activation by decreasing the expression levels of TLR4 proteins, NLRP3 inflammasome components (NLRP3, ASC, and caspase-1), GSDMD proteins, pro-inflammatory cytokines, such as IL-1β and IL-18, and LDH release. GSDMD, as a key executor in inflammatory caspase-induced pyroptosis (Kesavardhana & Kanneganti, 2017; Zhao Y. et al., 2018), is cleaved by caspase-1 at a specific site (the N-terminal domain of GSDMD), which binds to membrane lipid and lyses cells through pores with an inner diameter on the membrane, subsequently causing cell lysis and IL-1β release (DiPeso et al., 2017; Evavold et al., 2018;; Shi et al., 2015). A number of studies showed that NLRP3 inflammasome may play a vital role in the pathogenesis of active RA, including high expression of NLRP3 in the synovial tissue of collagen-induced arthritis (CIA) mice (Zhang Y. et al., 2016) and the peripheral blood cells of patients with active RA (Choulaki et al., 2015). Furthermore, the treatment with the NLRP3 selective inhibitor MCC950 exerted demonstrable pharmacodynamic effects on suppressing the NLRP3 inflammasome activation and amelioration of arthritic symptoms and cartilage destruction in CIA (Fan et al., 2016). Consistent with these previous studies, our results herein demonstrated that BHGZD may suppress the TLR4–mediated pyroptosis both *in vivo* and *in vitro* (Figure 6).

## Conclusion

The current study offers the experimental evidence that BHGZD may reverse the imbalance of the inflammation-immune system in the development and progression of RA *via* suppressing the TLR4–mediated NLRP3 inflammasome activation. These findings provide an in-depth understanding on the pharmacological mechanisms of BHGZD against RA, which may be of notable significance to the popularization of the application of TCM in the treatment of RA.

## Data Availability

Publicly available datasets were analyzed in this study. These data can be found here: GSE136098 (https://www.ncbi.nlm.nih.gov/geo/query/acc.cgi?acc = GSE136098).

## Abbreviations

9CK, creatine; AIA-M, adjuvant-induced arthritis model; ASC, apoptosis-associated speck-like protein containing a CARD; AST, aspartate aminotransferase; ATP, adenosine triphosphate; BHGZD, Baihu-Guizhi decoction; CFA, Freund’s complete adjuvant; c-Fos, Fos proto-oncogene, AP-1, transcription factor subunit; CIA, collagen-induced arthritis; CK-MB, isoenzyme; CRP, C-reactive protein; DAPI, 4′,6-diamidino-2-phenylindole; Difco, 1 mg of heat-killed *Mycobacterium tuberculosis* suspended in 0.1 ml paraffin oil; DMARDs, disease-modifying antirheumatic drugs; ESP, erythrocyte sedimentation rate; FBS, fetal bovine serum; GPI, glucose-6-phosphate isomerase; GSDMD, gasdermin-D; IDA, information-dependent acquisition; IL-1, interleukin 1; IL-17, interleukin 17; IL-18, interleukin 18; IL-1β, interleukin 1 beta; IL-2, interleukin 2; IL-22, interleukin 22; IL-35, interleukin 35; IL-6, interleukin 6; LDH, lactate dehydrogenase; LPS, lipopolysaccharide; MTX, methotrexate; NLRP3, NLR family pyrin domain containing 3; NSAIDs, nonsteroidal anti-inflammatory drugs; PI, propidium iodide; q-PCR, quantitative PCR detecting system; RA, rheumatoid arthritis; RF, rheumatoid factor; RT, retention time; TCM, traditional Chinese medicine; TLR4, toll-like receptor 4; TNF, tumor necrosis factor; TUNEL, terminal deoxynucleotidyl transferase–mediated dUTP-fluorescein nick end labeling; UFLC-Q-TOF-MS/MS, ultra-fast liquid chromatography-quadrupole-time-of-flight tandem mass spectrometry.
