# Supplementary material for: Disease-Modifying Anti-rheumatic Drug Prescription Baihu-Guizhi Decoction Attenuates Rheumatoid Arthritis via Suppressing Toll-Like Receptor 4-mediated NLRP3 Inflammasome Activation
Source: Front Pharmacol. 2021 Oct 5;12:743086. doi: 10.3389/fphar.2021.743086 (PMC8525175; doi:10.3389/fphar.2021.743086)
Supplement: Supplementary file 1 [file DataSheet1.docx]

**Supplementary Material for “Disease-Modifying Anti-rheumatic Drug Prescription Baihu-Guizhi Decoction Attenuates** **Rheumatoid Arthritis via Suppressing TLR4-mediated NLRP3 Inflammasome Activation”**

List of Supplementary Material:

Section 1 Preparation of Baihu-Guizhi decoction (BHGZD)

Section 2 Severity assessment of arthritis

Section 3 Measurement of the temperature of the articular surface in AIA-M rats

Section 4 Measurement of mechanical hypersensitivity

Section 5 Measurement of cold hypersensitivity

Section 6 Measurement of the heat hypersensitivity

Section 7 Indexes of liver, kidney, thymus and spleen

Table S1 The information on Enzyme-linked immunosorbent assay (ELISA) Kit

Fig.S1 Hepatotoxicity/nephrotoxicity assessment of Baihu-Guizhi decoction (BHGZD)

Fig. S2 Cytotoxicity of BHGZD on the growth of RAW264.7 macrophages cells

**Section 1 Preparation of Baihu-Guizhi decoction (BHGZD)**

The five traditional drugs [Gypsum (60g), Anemarrhena asphodeloides Bge. (15g), Cinnamomum cassia Presl (10g), Oryza sativa L. (30g), Glycyrrhiza uralensis Fisch. (5g)] were separately soaked with pure water for 1 hour. Then, Gypsum was added to 1200 mL (1: 10 g/v) of boiling pure water and boiled for 30 min. The herb mixture was added and boiled for 30 min. The filtrates were collected, and the residues were decocted in 600 mL (1: 5 g/v) of pure water for 20 min. The filtrates from each decoction were combined and concentrated to 2 g/mL using rotary evaporator. The obtained BHGZD was kept at -20℃ for preparation.

**Section 2 Severity assessment of arthritis**

Rats were observed once every day after primary immunization. Arthritis severity was evaluated by arthritis score, arthritis incidence, and percentage of arthritic limbs, which were performed by two independent, blinded observers. The arthritis score was the total of the scores for all 4 limbs (maximum possible arthritis score 80). Arthritis incidence values are the number positive/total number in group for 11 days consecutively. In addition, the number of arthritic limbs of individual rats were counted and added to represent the number of arthritic limbs in a group. The percentage of arthritic limbs in a group was calculated as following formula:

Eq. (A1) Percentage of arthritic limbs= $\frac{Number of arthritic limbs in a group}{Number of all limbs in a group}\times100\%$

Moreover, the time of arthritis first appeared referred to the first day of the onset of the clinical symptoms of arthritis observed.

**Section 3** **The temperature of the articular surface in AIA-M rats**

The temperature of the articular surface, awarded to the left hind paw of male Lewis rats, was measured using an Infrared thermal imager (TESTO-875, Testo AG, Schwarzwald, Germany) once a day from the day when the first signs of inflammatory were observed.

**Section 4 Measurement of mechanical-induced hyperalgesia**

Mechanical-induced hyperalgesia was assessed with Von Frey filaments by using Dixon’s up-and-down paradigm (Dixon, 1980). The rats were habituated in individual clear boxes on wire-mesh platform, to allow access to the ventral surface of the hind paws, and mechanical hypersensitivity was assessed by the sensitivity to the application of von Frey hairs (Stoelting Co., Chicago, USA). The von Frey filaments (1.4~180g) were presented perpendicularly to the plantar surface of the injected paw and held in this position for 2~3 s with enough force to cause a slight bend in the filament. Positive responses included an abrupt withdrawal of the hind paw or flinching behaviour immediately following removal of the stimulus, and 50% paw withdrawal threshold was determined at 0, 5, 10, 15, 25d after BHGZD administration (Wang et al., 2017), respectively.

**Section 5 Measurement of acetone-induced hypersensitivity**

The rats were acclimatized to an apparatus consisting of individual perspex boxes. With the aid of syringe (1 mL), 500 μL acetone was sprayed gently from a short distance to the middle of the plantar surface of hind paw. The acetone quickly spread over the proximal half of the plantar surface of the foot. The amount of time that rats were licking and/or shaking the hind paw was recorded during 5 min after acetone application and was used as an index of nocifensive responsiveness for cold hypersensitivity (Yoon, Wook, Sik, Ho, & Mo, 1994). The licking and/or shaking time was measured at 0, 5, 10, 15, 25 d after BHGZD administration, respectively.

**Section 6 Measurement of the thermal-induced hyperalgesia**

For thermal-induced hyperalgesia, the rats were placed for 30 minutes in an apparatus consisting of individual perspex boxes with Hargreaves radiant heat apparatus (Ugo Basile Srl, Comerio VA, Italy) and an infrared radiant heat (40 W) source was directed to middle part of the plantar surface of the hind paw. A cutoff time of 20 seconds was set to prevent tissue injury. The paw withdrawal latency was measured at 0, 5, 10, 15, 25 d after BHGZD administration, respectively. The interval of the paw withdrawal latency measurement was 5 min (Hargreaves, Dubner, Brown, Flores, & Joris, 1988).

**Section 7 Indexes of liver, kidney, thymus and spleen**

After sacrifice on the 28^th^ day of the experiments, the liver, kidney, thymus, spleen and brain were promptly removed and weighed. The indexes of these four organs were expressed as the ratio (mg/g) of liver, kidney, thymus, and spleen versus brain weight,

**Table S1**

**The information on Enzyme-linked immunosorbent assay (ELISA) Kit.**

| ELSIA Kit | Manufacturer | Lot No. |
| --- | --- | --- |
| Mouse IL-1β ELISA Kit | Shanghai Enzyme-linked Biotechnology Co., Ltd | ml063132-C |
| Mouse IL-18 ELISA Kit | Shanghai Enzyme-linked Biotechnology Co., Ltd | ml002294-C |
| Mouse LDH ELISA Kit | Shanghai Enzyme-linked Biotechnology Co., Ltd | ml002267-C |
| Human IL-1β ELISA Kit | Shanghai Enzyme-linked Biotechnology Co., Ltd | ml058059-C |
| Human IL-18 ELISA Kit | Shanghai Enzyme-linked Biotechnology Co., Ltd | ml058055-C |
| Human LDH ELISA Kit | Shanghai Enzyme-linked Biotechnology Co., Ltd | ml024518-C |
| Rat Toll-like receptor 4 (TLR4) ELISA Kit | Shanghai Enzyme-linked Biotechnology Co., Ltd | ml003365-C |
| Rat caspase-1 (Casp-1) ELISA Kit | Shanghai Enzyme-linked Biotechnology Co., Ltd | ml037348-C |
| Rat IL-1β ELISA Kit | Shanghai Enzyme-linked Biotechnology Co., Ltd | ml037361-C |
| Rat IL-18 ELISA Kit | Shanghai Enzyme-linked Biotechnology Co., Ltd | ml002816-C |
| Rat LDH ELISA Kit | Shanghai Enzyme-linked Biotechnology Co., Ltd | ml003416-C |


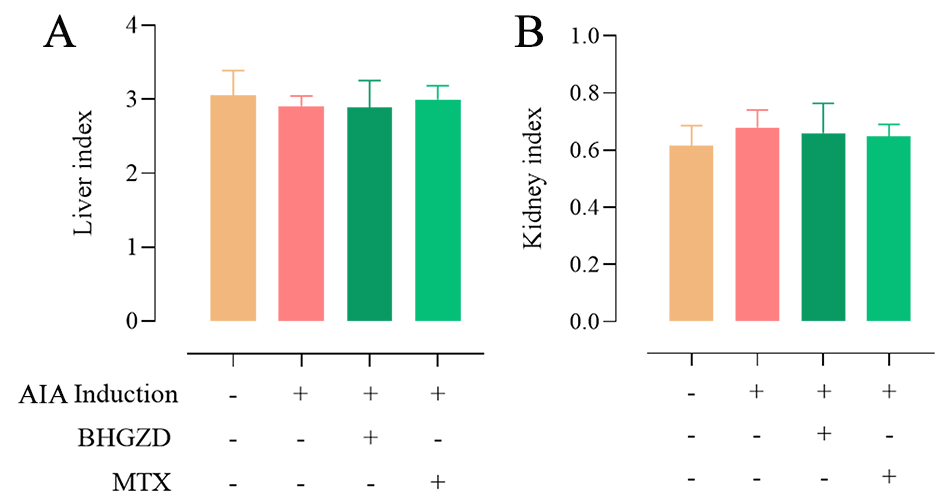


**Figure S1** Hepatotoxicity/nephrotoxicity assessment of Baihu-Guizhi decoction (BHGZD). (**A**) Liver index; (**B**) Kidney index.


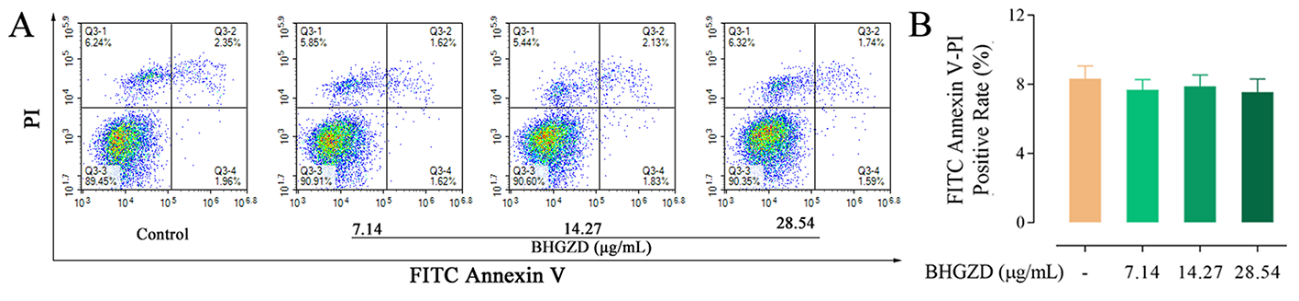


**Figure S2** Cytotoxicity of BHGZD on the growth of RAW264.7 macrophages cells. (**A~B**) flow cytometry analysis.

**References：**

Dixon, W. J. (1980). Efficient analysis of experimental observations. *Annu Rev Pharmacol Toxicol, 20*, 441-462. doi:10.1146/annurev.pa.20.040180.002301

Hargreaves, K., Dubner, R., Brown, F., Flores, C., & Joris, J. (1988). A new and sensitive method for measuring thermal nociception in cutaneous hyperalgesia. *Pain, 32*(1), 77-88. doi:10.1016/0304-3959(88)90026-7

Wang, C., Kong, X., Zhu, C., Liu, C., Sun, D., Xu, Q., Mao Z., Qian Q., Su H., Wang D., Zhao X., Lin N. (2017). Wu-tou decoction attenuates neuropathic pain via suppressing spinal astrocytic IL-1R1/TRAF6/JNK signaling. *Oncotarget, 8*(54), 92864-92879. doi:10.18632/oncotarget.21638

Yoon, C., Wook, Y. Y., Sik, N. H., Ho, K. S., & Mo, C. J. (1994). Behavioral signs of ongoing pain and cold allodynia in a rat model of neuropathic pain. *Pain, 59*(3), 369-376. doi:10.1016/0304-3959(94)90023-X
